# Loss of FBXO7 results in a Parkinson's‐like dopaminergic degeneration via an RPL23–MDM2–TP53 pathway

**DOI:** 10.1002/path.5312

**Published:** 2019-08-06

**Authors:** Simon RW Stott, Suzanne J Randle, Sara Al Rawi, Paulina A Rowicka, Rebecca Harris, Bethany Mason, Jing Xia, Jeffrey W Dalley, Roger A Barker, Heike Laman

**Affiliations:** ^1^ John van Geest Centre for Brain Repair University of Cambridge Cambridge UK; ^2^ Department of Pathology University of Cambridge Cambridge UK; ^3^ Behavioural and Clinical Neuroscience Institute and Department of Psychology University of Cambridge Cambridge UK; ^4^ Department of Psychiatry University of Cambridge Cambridge UK; ^5^ Wellcome – MRC Stem Cell Institute University of Cambridge Cambridge UK

**Keywords:** *FBXO7/PARK15*, Parkinson's disease, dopaminergic neurons, Rpl23–Mdm2–p53 axis

## Abstract

The field of Parkinson's disease research has been impeded by the absence of animal models that clearly phenocopy the features of this neurodegenerative condition. Mutations in *FBXO7/PARK15* are associated with both sporadic Parkinson's disease and a severe form of autosomal recessive early‐onset Parkinsonism. Here we report that conditional deletion of *Fbxo7* in the midbrain dopamine neurons results in an early reduction in striatal dopamine levels, together with a slow, progressive loss of midbrain dopamine neurons and onset of locomotor defects. Unexpectedly, a later compensatory response led to a near‐full restoration of dopaminergic fibre innervation in the striatum, but nigral cell loss was irreversible. Mechanistically, there was increased expression in the dopamine neurons of FBXO7‐interacting protein, RPL23, which is a sensor of ribosomal stress that inhibits MDM2, the negative regulator of p53. A corresponding activated p53 transcriptional signature biased towards pro‐apoptotic genes was also observed. These data suggest that the neuroprotective role of FBXO7 involves its suppression of the RPL23–MDM2–p53 axis that promotes cell death in dopaminergic midbrain neurons. © 2019 The Authors. *The Journal of Pathology* published by John Wiley & Sons Ltd on behalf of Pathological Society of Great Britain and Ireland.

## Introduction

Parkinson's disease (PD) is characterised by the aggregation of alpha‐synuclein and loss of specific neurons in the brain, which is accompanied by a defined set of clinical features, including bradykinesia, akinesia, and a resting tremor 1. Numerous genetic variations have been associated with PD 2, but few of the transgenic mice that have been generated incorporating these mutations have resulted in a phenocopy of the condition 3. FBXO7 (also known as PARK15) belongs to the F‐box protein family which recruits substrates to SCF‐type E3 ubiquitin ligases. Because F‐box proteins act at the penultimate step in ubiquitin transference, they are regarded as hubs in ubiquitin signalling. FBXO7 also has ubiquitin‐independent functions, including binding other PD‐associated proteins, PARKIN and PINK1, to regulate mitophagy, and binding the G1 kinase, CDK6, and cell cycle inhibitor p27 to regulate the cell cycle 4.

In 2008, a point mutation in *FBXO7* was linked to a familial form of PD 5, presenting an early‐onset Parkinsonism with pyramidal signs. Subsequently additional mutations have been found in early‐onset cases and in patients with sporadic PD 6, 7, 8, 9, 10, 11, 12. PARK15 PD patients are levodopa‐responsive, but rapidly develop side effects, such as dyskinesia 11, 12. To date, a neuropathological analysis of the brain of an individual with *FBXO7*‐associated PD has not been reported. However, brain imaging (DaTSCAN‐SPECT) suggests that there is a significant reduction in the dopaminergic fibre innervation of the putamen 12. Whether there is any loss of dopamine (DA) neurons in the substantia nigra is not known.

FBXO7 acts in a PARKIN‐mediated pathway to regulate mitophagy. The disease‐associated T22M mutation in FBXO7 compromises PARKIN binding, supporting the idea that loss of their interaction is pathological in humans 13. However, evidence from a *Drosophila* model of neurodegeneration caused by Parkin loss indicates that FBXO7's canonical ubiquitin ligase activity is also critical for neuronal health. Expression of FBXO7 rescues motor deficits of *parkin* ^*−/−*^ flies, arguing that FBXO7 can substitute for Parkin‐mediated ubiquitination of mitochondrial substrates. Moreover, the expression of pathological alleles of FBXO7 that compromise its ubiquitin‐ligase activity or substrate recruitment was unable to rescue *parkin* ^*−/−*^ defects. These findings argue that FBXO7 recruitment of PARKIN and its ubiquitination of substrates mediate its neuro‐protective properties.

As FBXO7 is a multifunctional protein with distinct cellular activities, investigating its activity in neurons is necessary to determine its functions and how it contributes to PD. To test the requirements of *Fbxo7* in dopaminergic neurons, we utilised mouse models of *Fbxo7* loss: a complete null (*Fbxo7* ^*−/−*^) and a conditional loss of *Fbxo7* in DA neurons expressing the dopamine transporter (*Dat*). Our results indicate the loss of Fbxo7 in midbrain dopaminergic cells leads to a reduction of synaptic DA release in the striatum and to locomotor defects. Within the midbrain, we also found a slow loss of DA cells and activation of the stress‐responsive RPL23–MDM2–p53 pathway.

## Materials and methods

### Mice

All experiments in mice were performed in accordance with the UK Animals (Scientific Procedures) Act 1986 and ARRIVE guidelines. Animal licences were approved by the Home Office and the University of Cambridge's Animal Welfare & Ethical Review Body Standing Committee. Experiments were performed under Home Office licences PPL 80/2474, 70/9001, 80/2366, and 70/8411. All mice were bred as heterozygous crosses, and both male and female mice were used in experiments. *Fbxo7* ^*LacZ*^ mice (*Fbxo7* ^*tm1a(EUCOMM)Hmgu*^ on a C57BL/6J background) were crossed to ActB:FLPe animals 14, then to either *ZP3* ^Cre^ 15 or *Dat* ^*Cre*^ *ROSA26*‐stop‐*YFP* animals 16, to generate complete or conditional null mice, respectively.

### Behavioural testing

Mice were tested using rotarod and open field locomotion assessments. Details are available in supplementary material, Supplementary materials and methods.

### Tissue processing

For HPLC and immunohistochemistry analysis, brains were snap‐frozen and dissected from cryostat‐cut 30‐μm‐thick coronal sections. Details are available in supplementary material, Supplementary materials and methods. For other experiments, mice were euthanized with a 0.5 ml intraperitoneal injection of Euthatal (pentobarbitone sodium, 200 mg/ml; Merial Animal Health Ltd, Boehringer Ingelheim Animal Health, Bracknell, Berkshire, UK) and perfused transcardially with 0.9% saline followed by ice‐cold 4% paraformaldehyde (0.1 m phosphate buffer, pH 7.4). Brains were removed and post‐fixed in 4% PFA overnight, and then placed in 30% sucrose.

### Stereological analysis

Randomised estimations of the total number of TH^+^ cells in the ventral midbrain of the mice were performed using a standard stereological method (Olympus CAST Grid System). Details are available in supplementary material, Supplementary materials and methods.

### Optical density analysis and cell body size measurements

To determine the fibre density, the mean optical intensity was measured from the TH^+^ stained sections. The area of the soma of SNpc TH^+^ neurons was measured (*n* = 3 per genotype; minimum 200 cells per brain) from fluorescent images of TH^+^ neurons. Details are available in supplementary material, Supplementary materials and methods.

### Neurotransmitter measurements

DA and norepinephrine were measured using reversed‐phase high performance liquid chromatography (HPLC) and electrochemical detection. Details are available in supplementary material, Supplementary materials and methods.

### Tissue and cell lysis, immunoprecipitation, and immunoblotting

Frozen brain regions were lysed in RIPA buffer (10 μl/mg tissue) containing protease and phosphatase inhibitors, and samples homogenised using a Dounce homogeniser. For transfected HEK293T and SHSY‐5Y experiments, cells were lysed in RIPA or NETN buffer, and 50 μg of the lysate was analysed by immunoblotting or subjected to immunoprecipitation. Details are available in supplementary material, Supplementary materials and methods.

### Isolation of RNA and RT‐qPCR

Ventral midbrain regions (∼10 mg) were micro‐dissected. Total RNA was isolated using an RNeasy Plus kit (Qiagen, Manchester, UK), and converted to cDNA using Quantitect Reverse Transcriptase (Qiagen) and then diluted 1:10 for subsequent qPCR analysis using SYBR Green JumpStart Taq (Sigma Aldrich, Gillingham, UK) on a CFX Connect Real‐Time PCR machine (Bio‐Rad, Watford, UK). Details are available in supplementary material, Supplementary materials and methods.

## Results

### *Fbxo7* mRNA is widely expressed in the adult mouse brain

**Figure 1:**
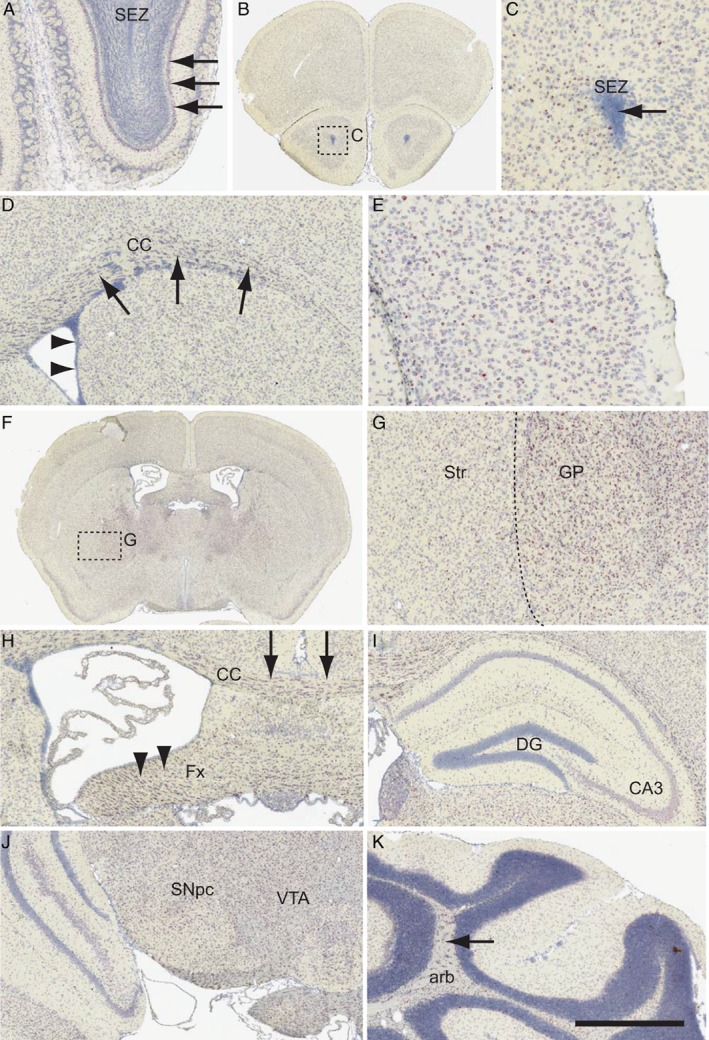
*In situ* hybridisation for *Fbxo7* in the adult mouse brain. *Fbxo7* transcripts were labelled using branched DNA amplification *in situ* hybridisation. The regions shown include (A) olfactory bulb, (B, C) the subependymal zone (SEZ) of the rostral forebrain, (D) the corpus callosum (CC), (E) the overlying cortex, (F, G) the striatum (Str) and globus pallidus (GP), (H) the fornix (Fx), (I) the dentate gyrus (DG) and CA3 region of the hippocampus, (J) the substantia nigra pars compacta (SNpc) and ventral tegmental area (VTA) of the ventral midbrain, and (K) the arbor vitae (arb) of the cerebellum. The scale bar in K represents 300 μm for A, D, H, I, J; 200 μm for C, E; 2 mm for B, F; 250 μm for G; and 600 μm for K.

We analysed *Fbxo7* mRNA expression in the adult mouse brain using *in situ* hybridisation. Widespread, low‐level expression of *Fbxo7* transcripts were seen throughout the brain, with higher levels in the mitral cell layer of the olfactory bulb (OB) (arrows; Figure 1A). *Fbxo7* transcripts were absent from the subependymal zone (SEZ) in the OB and throughout the forebrain (arrowheads; Figure 1D), although cells immediately adjacent to the SEZ in the anterior olfactory nucleus were positive (Figure 1B,C). In the striatum, *Fbxo7* expression was present in the corpus callosum (CC), possibly in oligodendrocytes (arrows; Figure 1D). There was very limited expression in the striatum (Str) (Figure 1D), compared with a ‘salt and pepper’ pattern in overlying cortex (Figure 1E). The lower expression of *Fbxo7* in the striatum was particularly apparent when comparing the Str with the adjacent globus pallidus (GP) (Figure 1F,G). At this same level, there were many labelled transcripts in the CC and fornix (Fx) (arrows and arrowheads respectively; Figure 1H). Expression in the hippocampus was limited to scattered sparse labelling in the CA3 region of the hippocampus (Figure 1I). More caudally, the majority of nuclei in the midbrain region were positive for *Fbxo7* mRNA, including the dopamine (DA) neurons of the substantia nigra pars compacta (SNpc) and ventral tegmental area (VTA; Figure 1J). Ubiquitous *Fbxo7* mRNA expression in the midbrain was evident also in the hindbrain, where it was present in most regions except for the SEZ (data not shown). In the cerebellum, *Fbxo7* expression was strongest in the arbor vitae (arb; arrow), with little labelling in the granular or Purkinje cell layers (Figure 1K). Overall, the expression of *Fbxo7* mRNA in the adult mouse brain was less than that of control genes (e.g. *Cd24* 17), but appeared to be strongest in the white matter regions and the evolutionarily older structures, such as the midbrain and brain stem.

In our analyses of FBXO7 protein in mouse brain, none of the antibodies that we tested gave a reliable and specific signal with IHC compared with *Fbxo7* null animals; therefore, we dissected out regions of the brain and performed immunoblotting on protein lysates to verify FBXO7 expression. We detected expression in all regions of the brain analysed, with the highest levels being found in the OB (data not shown). FBXO7 isoform 1 was expressed, but murine isoform 2, which migrates at ∼55 kDa, was not detected. These data demonstrate widespread FBXO7 expression throughout the brain of adult mice.

### *Fbxo7* null mice fail to thrive

**Figure 2:**
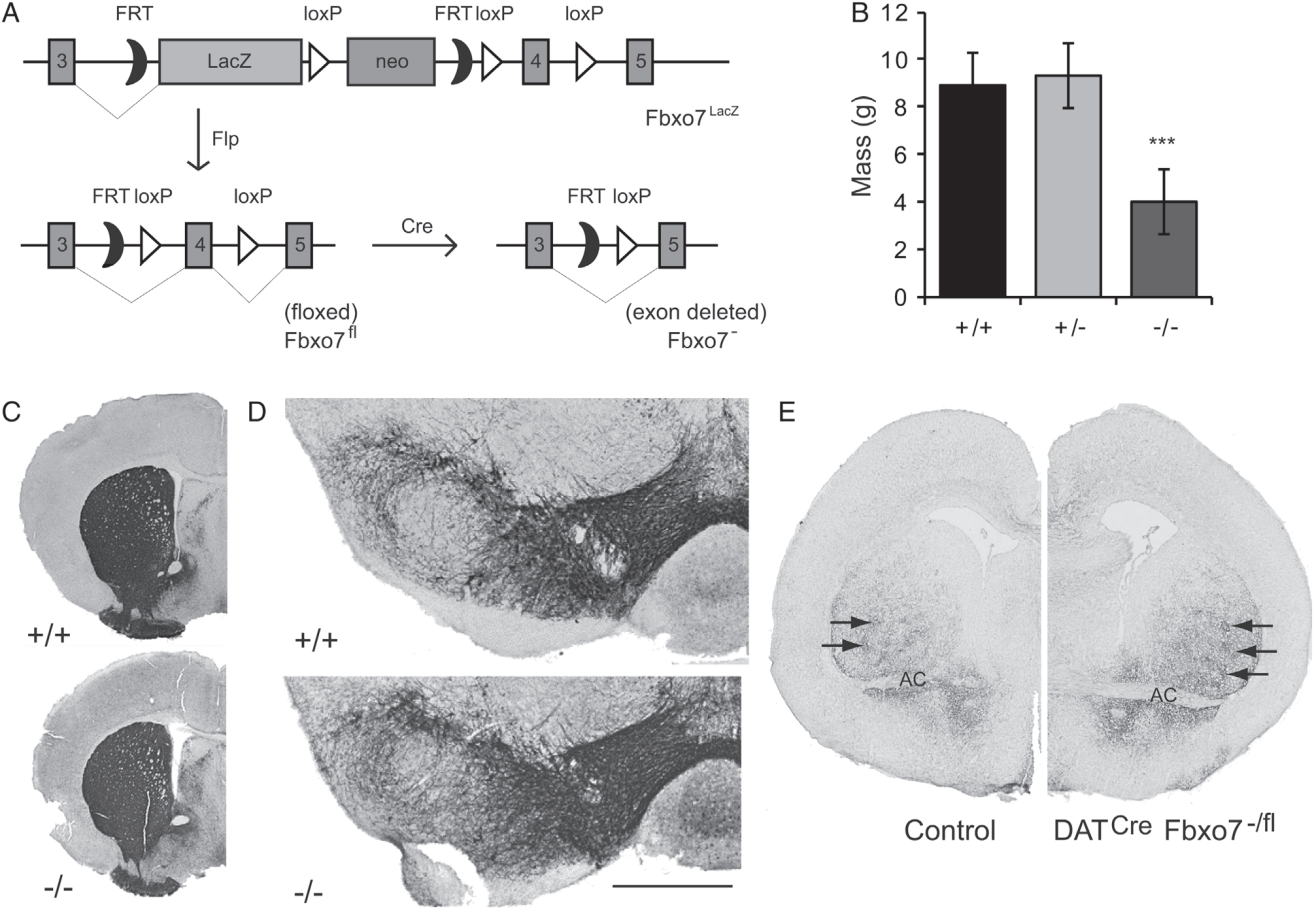
*Fbxo7* null mice fail to thrive. (A) Schematic diagram of the transgene used to generate various mice. (B) Body weight measurements of WT, heterozygous *Fbxo7* ^−/+^, and *Fbxo7* ^−/−^ mice at P18. (C) Representative TH‐immunostained sections from WT and *Fbxo7* ^−/−^ mice at the level of the striatum. (D) Representative TH‐immunostained sections from WT and *Fbxo7* ^−/−^ mice at the level of the SNpc. (E) TH staining of the developing striatum at birth, at the level of the anterior commissure (AC), in the *Dat* ^*Cre*^ *Fbxo7* ^−/+^ (control) and *Dat* ^*Cre*^ *Fbxo7* ^−/fl^ mice, demonstrating the presence of striosomes (arrows). Scale bar in D represents 2 mm for C, 500 μm for D, and 1 mm for E.

To determine functional roles *in vivo*, we created *Fbxo7* null mice, first by crossing *Fbxo7* ^*LacZ*^ mice 18, 19 with ActB:FLPe animals 14, to generate a ‘floxed’ (*Fbxo7* ^fl^) allele, with LoxP sites flanking either side of exon 4 (Figure 2A), and subsequent breeding to *Zp3* ^*Cre*^ mice 15, for germline excision (*Fbxo7* ^−^). *Fbxo7* ^−/+^ offspring were backcrossed with WT C57/BL6J mice and bred to homozygosity. *Fbxo7* null mice (*Fbxo7* ^−/−^) appeared normal at birth, but by postnatal day 10 (P10) showed a significant growth defect (see supplementary material, Figure S1A), despite evidence of feeding. By P18, *Fbxo7* ^−/−^ mice were, on average, 45% of the body mass of WT and *Fbxo7* ^−/+^ littermates (Figure 2B; *n* = 7), with a runty, hunched appearance. Mice failed to survive post‐weaning. No evidence of infection was detected, either histologically or serologically (data not shown). Histological analyses of major organs were unremarkable, and analyses of brain regions showed normal cellularity and no gross anatomical defects (data not shown). Dopaminergic fibre density in the striatum was indistinguishable from WT mice [based on optical density: the *Fbxo7* ^−/−^ striatum was 99.3 ± 2.3% of WT; *n* = 4 in both groups (Mann–Whitney *U*‐test (M‐W U) = 0.0, *p* = 0.82), and the *Fbxo7* ^−/−^ nucleus accumbens was 100.7 ± 2.4% of WT; *n* = 4 in both groups (M‐W U = 0.0, *p* = 0.73; Figure 2C)] and a normal population of tyrosine hydroxylase (TH) positive neurons was present in the substantia nigra (Figure 2D and Table 1). These data demonstrate that the expression of FBXO7 is required for post‐weaning survival.

**Table 1:** Stereological estimations of TH^+^ cells in the midbrain of the mice used in this study, in the substantia nigra (SN), ventral tegmental area (VTA), and the striatum

Age	Genotype	*N*	Average number TH^+^ cells (± SEM)	Significance
SN stereological estimations				
3 weeks	Fbxo7^−/−^	4	9601.3 ± 316.8	
	Fbxo7^−/+^	5	9849.1 ± 406.4	
	Fbxo7^+/+^	2	10 517.1 ± 63.3	F(2,9) = 2.101, *p* = 0.18
18 months	Fbxo7^LacZ/LacZ^	3	10 081.5 ± 339.5	
	Fbxo7^LacZ/+^	5	9468.5 ± 207.8	
	Fbxo7^+/+^	4	9790.5 ± 280.4	F(2,10) = 0.087, *p* = 0.91
6 weeks	*Dat* ^*Cre*^; Fbxo7^−/fl^	3	8116.9 ± 452.9	
	Controls	9	8732.3 ± 279.9	M‐W U = 4, *p* = 0.091
12 weeks	*Dat* ^*Cre*^; Fbxo7^−/fl^	5	7968.4 ± 236.2	
	Controls	6	8825.6 ± 69.1	M‐W U = 7, *p* = 0.106
20 weeks	*Dat* ^*Cre*^; Fbxo7^−/fl^	4	7739.3 ± 214.4	
	Controls	6	9201.1 ± 265.8	M‐W U = 5, *p* = 0.016
46 weeks	*Dat* ^*Cre*^; Fbxo7^−/fl^	6	8557.3 ± 304.1	
	Controls	8	9390.9 ± 310.9	M‐W U = 0, *p* = 0.016
VTA stereological estimations				
46 weeks	*Dat* ^*Cre*^; Fbxo7^−/fl^	6	9462.2 ± 351.6	
	Controls	8	10 797.8 ± 125.7	M‐W U = 5, *p* = 0.013
Striatal stereological estimations				
6 weeks	*Dat* ^*Cre*^; Fbxo7^−/fl^	3	10 937.5 ± 1701.3	
12 weeks	*Dat* ^*Cre*^; Fbxo7^−/fl^	5	20 937.5 ± 2610.7	
20 weeks	*Dat* ^*Cre*^; Fbxo7^−/fl^	4	23 531.3 ± 3402.8	
46 weeks	*Dat* ^*Cre*^; Fbxo7^−/fl^	6	29 390.63 ± 1297.1	F(2,11) = 8.329, *p* = 0.0006
46 weeks	*Dat* ^*Cre*^; Fbxo7^−/fl^	3	1 039 416.67 ± 123 668.1 (NeuN^+^ cells)	

The number of NeuN^+^ cells was also calculated to determine the neuronal population of the region. ANOVA and Mann–Whitney (M‐W) statistical results are presented.

### Conditional *Fbxo7* knockout in dopaminergic neurons leads to a reduction in dopamine release in the striatum

To generate a mouse in which *Fbxo7* was lost specifically in dopaminergic neurons, *Fbxo7* ^fl^ mice were bred with *Dat* ^*Cre*^ *ROSA26*‐stop‐*YFP* animals, which express Cre from the dopamine transporter (*Dat*) promoter 16. Since our previous studies detected no phenotypes in mice heterozygous for *Fbxo7* 18, 19, we analysed *Dat* ^*Cre*^ *Fbxo7* ^−/fl^ animals, which were systemically heterozygous for Fbxo7 expression, in addition to the loss of Fbxo7 expression in dopaminergic cells. *Dat* ^*+/+*^ *Fbxo7* ^−/+^ and *Dat* ^*Cre*^ *Fbxo7* ^−/+^ mice were used as controls.

**Figure 3:**
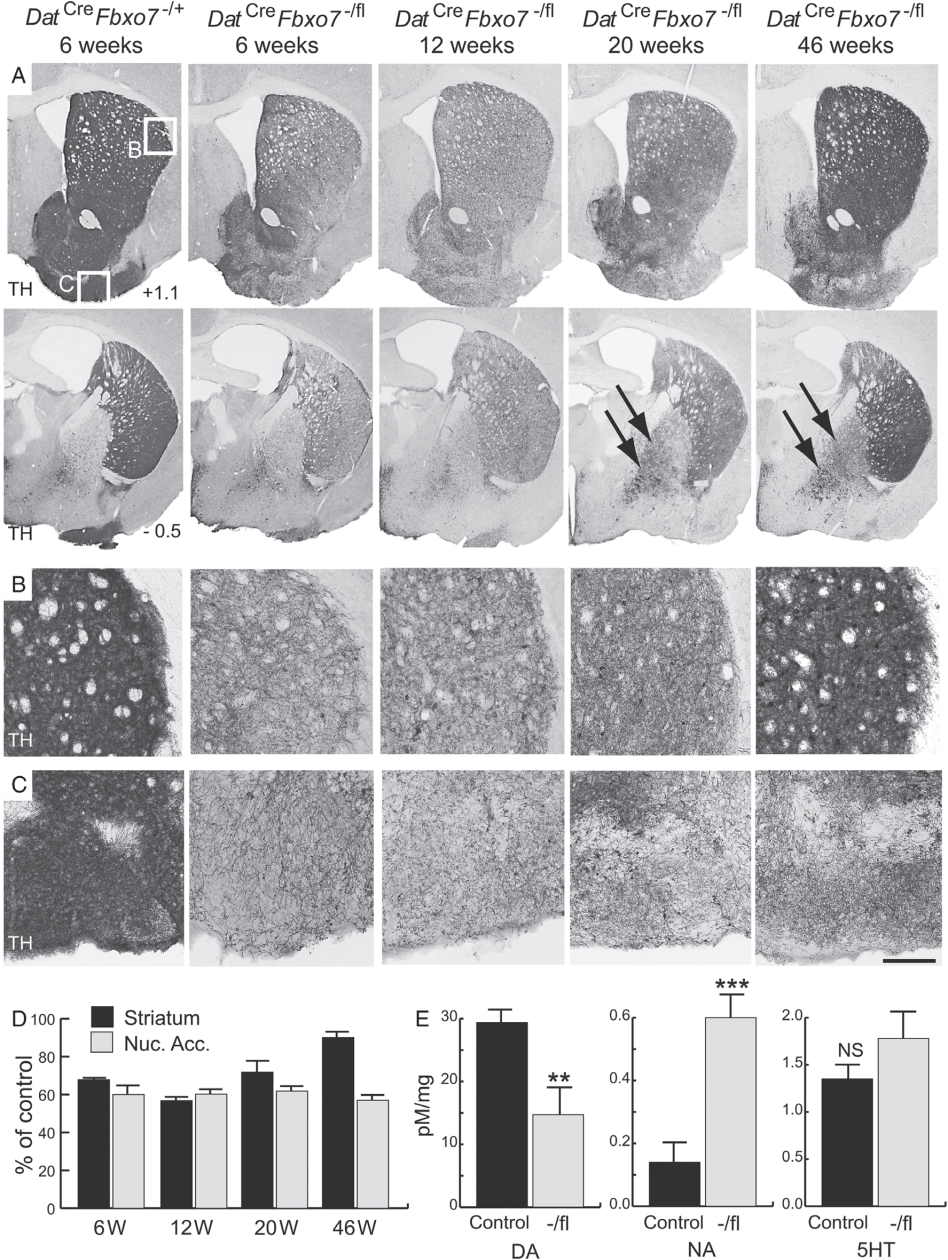
Decreased TH^+^ staining in mice with a conditional loss of *Fbxo7* expression in dopaminergic cells. (A) TH‐immunostained sections of the striatum at +1.1 mm (top panels) and −0.5 mm (bottom panels) relative to the bregma from control mice (*Dat* ^*Cre*^ *Fbxo7* ^−/+^) at 6 weeks, and conditional KO mice (*Dat* ^*Cre*^ *Fbxo7* ^−/fl^) at 6, 12, 20, and 46 weeks of age. Arrows show increased TH staining in the globus pallidus over time. (B, C) Magnified regions highlighted in A, showing TH^+^ fibres in the striatum (B) and olfactory tubercle (C). (D) Quantification of TH^+^ fibre density in *Dat* ^*Cre*^ *Fbxo7* ^−/fl^ mice at different time points, as a percentage of control at each time point, in the striatum and nucleus accumbens (Nuc. Acc.). (E) Quantification of neurotransmitter levels in the striatum of 5‐week‐old *Dat* ^*Cre*^ *Fbxo7* ^−/+^ and *Dat* ^*Cre*^ *Fbxo7* ^fl/+^ mice (control) and *Dat* ^*Cre*^ *Fbxo7* ^−/fl^ mice (−/fl) using HPLC analysis. DA, dopamine; NA, noradrenaline; 5HT, serotonin. **p < 0.01; ***p < 0.001. NS, not significant. The scale bar in C represents 800 μm for A and 300 μm for B, C.

In *Dat* ^*Cre*^ mice, Cre‐mediated recombination in DA neurons initiates around E13 20. TH^+^ fibre innervation of the striatum begins at approximately E16 and continues until 8–9 weeks, when striatal DA levels plateau 21. At birth (P0), *Dat* ^*Cre*^ *Fbxo7* ^−/fl^ mice had a normal distribution of DA neurons in the ventral midbrain (VM) and axonal fibres in the lateral ventral portion of the developing striatum (Figure 2E). TH^+^ striosomes, as determined by double labelling with μ‐opioid staining (data not shown), were clearly visible. We also confirmed that TH^+^ neurons in the VM of *Dat* ^*Cre*^ *Fbxo7* ^−/fl^ animals expressed Cre (see supplementary material, Figure S1B). At 6 weeks, control *Dat* ^*Cre*^ *Fbxo7* ^−/+^ mice showed robust TH expression uniformly present across the whole of the striatal region (Figure 3A), with magnified striatum (Figure 3B) and olfactory tubercle (Figure 3C). In contrast, *Dat* ^*Cre*^ *Fbxo7* ^−/fl^ mice exhibited significantly less TH expression in the striatum (69.64 ± 1.9% of control; *n* = 3; M‐W U = 0.0, *p* = 0.024; Figure 3A–D). Dense clustering of TH^+^ fibres was apparent close to the ventricular space and around striosomes but was reduced elsewhere. This reduced TH^+^ fibre density in the striatum was supported by neurotransmitter measurements showing a 50.2 ± 4.5% reduction in striatal DA levels in *Dat* ^*Cre*^ *Fbxo7* ^−/fl^ mice, alongside a 428.5 ± 51.5% increase in noradrenaline levels (Figure 3E). We assayed later time points to determine whether this phenotype progressed or was corrected. At 12 weeks, by which time striatal TH^+^ fibre density and DA levels should have plateaued, *Dat* ^*Cre*^ *Fbxo7* ^−/fl^ mice presented a 44.4 ± 1.7% (*n* = 3; M‐W U = 0.0, *p* = 0.023) and a 40.9 ± 2.9% (M‐W U = 0.0, *p* = 0.024) decrease in the density of TH^+^ fibres in the striatum and nucleus accumbens, respectively, compared with controls (Figure 3A–D). Remarkably, by 20 weeks there was an *increase* in TH^+^ fibre density in the striatum of *Dat* ^*Cre*^ *Fbxo7* ^−/fl^ mice compared with that seen at 12 weeks, but not in the nucleus accumbens [28.7 ± 6.2% decrease in TH^+^ fibres in the striatum (*n* = 4; M‐W U = 0.0, *p* = 0.003) versus a 47.5 ± 2.0% reduction in the nucleus accumbens (M‐W U = 0.0, *p* = 0.002); Figure 3A–D]. This trend continued to 46 weeks of age when the striatum exhibited a near‐complete restoration of TH^+^ fibre density levels, while the nucleus accumbens displayed no change [13.0 ± 3.8% reduction in the striatum (*n* = 6; M‐W U = 5, *p* = 0.013) versus a 42.7 ± 2.0% reduction in the nucleus accumbens (M‐W U = 0, *p* = 0.0007); Figure 3A–D]. At both the 20‐ and the 46‐week time points, there was also increased TH^+^ staining in the globus pallidus (arrows in Figure 3A). Collectively, these data indicate that loss of Fbxo7 in DA neurons results in an abnormal presentation of the midbrain dopamine system at the level of the striatum.

### Ectopic expression of TH in the striatum of the conditional *Fbxo7* knockout mouse

**Figure 4:**
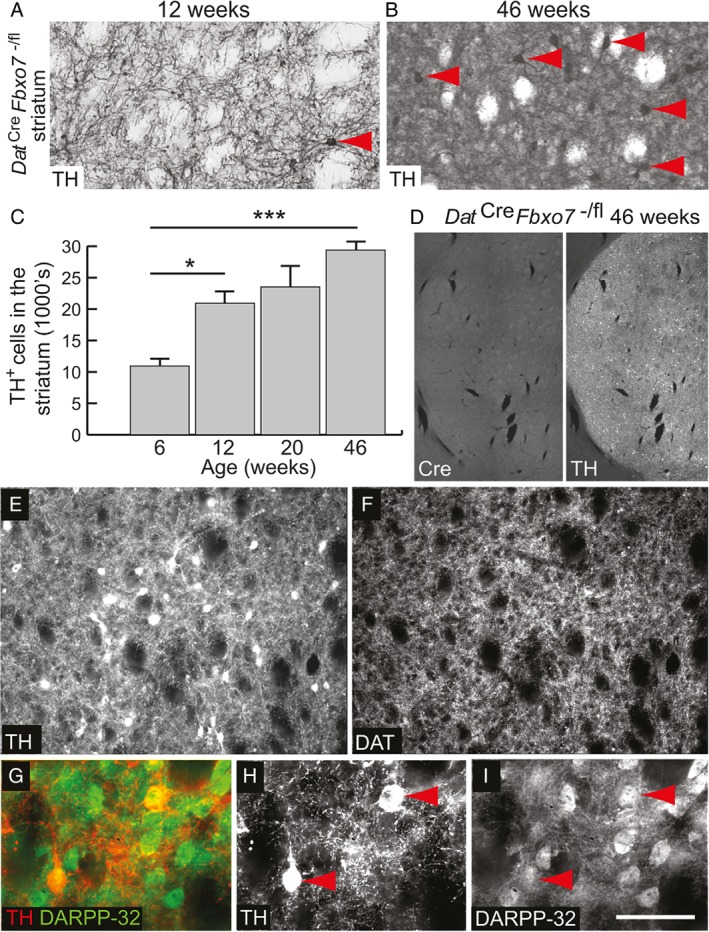
Ectopic expression of TH in the striatum of mutant mice. (A, B) Magnified image of TH‐immunostained striatum from *Dat* ^*Cre*^ *Fbxo7* ^−/fl^ mice at (A) 12 weeks and (B) 46 weeks, showing TH^+^ cell bodies (red arrowheads). (C) Quantification of TH^+^ cell bodies in the striatum. ANOVA *p < 0.05; ***p < 0.001. (D) Immunofluorescence staining for Cre and TH in the striatum from *Dat* ^*Cre*^ *Fbxo7* ^−/fl^ mice, demonstrating no ectopic Cre expression. (E, F) Immunofluorescence staining for TH (E) and DAT (F) in the striatum of 46‐week‐old *Dat* ^*Cre*^ *Fbxo7* ^−/fl^ mice. TH^+^ cell bodies in the striatum are negative for DAT expression. (G–I) Immunofluorescence staining for (H) TH and (I) DARPP‐32 with (G) merged image in the striatum of 46‐week‐old *Dat* ^*Cre*^ *Fbxo7* ^−/fl^ mice. TH^+^ cell bodies in the striatum are positive for DARPP‐32 expression (red arrowheads), suggesting medium spiny neuron origin. The scale bar in I represents 300 μm for A, B, E, F; 1 mm for D; and 100 μm for G–I.

**Figure 5:**
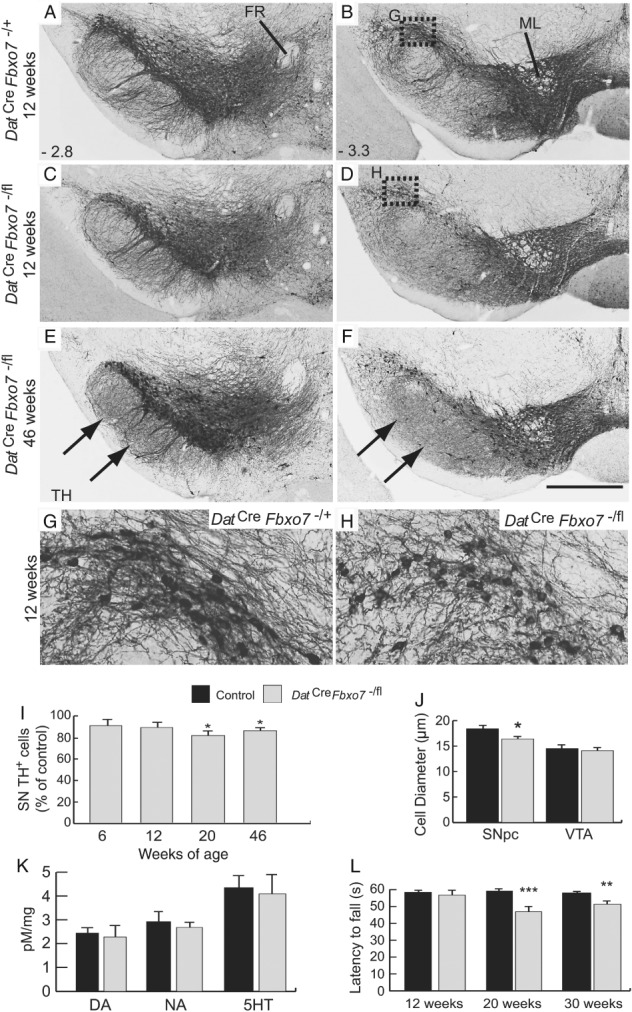
Smaller cell size and decreased cell number in the substantia nigra of mutant mice. (A–F) TH immunostaining of the VTA and substantia nigra from (A, B) control and (C–F) mutant mice at 12 (A–D) and 46 weeks (E, F) at −2.8 mm (A, C, E) and −3.3 mm (B, D, F) relative to the bregma. Arrows in E and F highlight regions of increased TH^+^ expression in the very fine fibres of the striatonigral pathway in the SNpr (originating from TH^+^ cells in the striatum). (G, H) Magnification of the SNpc regions highlighted in B and D, of control (G) and mutant (H) mice, showing smaller TH^+^ cell bodies and fewer neurites in mutant mice. (I) Stereological estimations of TH^+^ cell bodies in the SNpc of *Dat* ^*Cre*^ *Fbxo7* ^−/+^ and *Dat* ^*Cre*^ *Fbxo7* ^fl/+^ mice (control), and experimental *Dat* ^*Cre*^ *Fbxo7* ^−/fl^ mice at 6, 12, 20, and 46 weeks. (J) Quantification of the diameter of TH^+^ cell bodies in the SNpc and VTA of control and *Dat* ^*Cre*^ *Fbxo7* ^−/fl^ mice at 12 weeks. (K) Quantification of neurotransmitter levels in the ventral midbrain of 5‐week‐old *Dat* ^*Cre*^ *Fbxo7* ^−/+^ and *Dat* ^*Cre*^ *Fbxo7* ^fl/+^ mice (control) and *Dat* ^*Cre*^ *Fbxo7* ^−/fl^ mice using HPLC analysis. (L) Rotarod behavioural analysis of control and *Dat* ^*Cre*^ *Fbxo7* ^−/fl^ mice at 12, 20, and 30 weeks of age, showing latency to fall. **p < 0.05; ***p < 0.005. FR, fasciculus retroflexus; ML, medial lemniscus; DA, dopamine; NA, noradrenaline; 5HT, serotonin. The scale bar in F represents 300 μm for A–F and 80 μm in G, H.

Closer examination of the re‐establishment of TH^+^ fibre density in the striatum between 12 and 46 weeks of age in the *Dat* ^*Cre*^ *Fbxo7* ^−/fl^ mice revealed the presence of large TH^+^ cell bodies (arrowheads in Figure 4A,B). Quantification of these TH^+^ cell bodies in the striatum indicated an increasing trend across all of the time points examined, representing almost 3% of the NeuN^+^ neurons in the striatum by 46 weeks (Figure 4C). There was no corresponding increase in Cre expression in these cells, suggesting that no ectopic allelic recombination was occurring (Figure 4D). In addition, the TH^+^ cell bodies in the striatum did not express DAT (Figure 4E,F). Instead, the majority of these ectopically expressing TH^+^ cell bodies in the striatum were identified as medium spiny projection neurons due to co‐expression of DARPP‐32 (arrowheads in Figure 4H,I). This finding explains the increase in TH^+^ fibre staining observed in the globus pallidus (arrows in Figure 3A) and SNpr of the 46‐week‐old *Dat* ^*Cre*^ *Fbxo7* ^−/fl^ mice, as the striatopallidal and striatonigral pathways of these mice were now ectopically expressing TH (arrows in Figure 5E,F). These data also suggest that a proportion of the re‐innervation of the striatum can be attributed to ectopic expression of TH in the *Dat* ^*Cre*^ *Fbxo7* ^−/fl^ mice, which may be a compensatory mechanism, similar to those noted in previous mouse, rat, and primate models of DA loss 21, 22, 23.

### Delayed loss of dopamine neurons in the conditional *Fbxo7* knockout

Despite the significant reduction in striatal TH^+^ fibres, there was no significant difference in the number of DA neurons in the SNpc between *Dat* ^*Cre*^ *Fbxo7* ^−/fl^ mice and control littermates at 6 and 12 weeks (Figure 5A–D,I and Table 1). Consistent with this, there was no difference in neurotransmitter levels in the VM of *Dat* ^*Cre*^ *Fbxo7* ^−/fl^ mice (Figure 5K). However, we observed a significant difference in the diameter of TH^+^ cell bodies in the SNpc at 12 weeks of age (88.9 ± 3.1%; M‐W U = 13, *p* = 0.012; Figure 5G,H,J) but not in the VTA (97.4 ± 1.2%; M‐W U = 63, *p* = 0.79; Figure 5J). There was also a noticeable reduction in the density of neurites from each SNpc TH^+^ cell at 12 weeks (Figure 5G,H). This shrinkage and reduction in neurites of the TH^+^ SNpc cells at 12 weeks preceded a significant reduction in the number of TH^+^ cells in *Dat* ^*Cre*^ *Fbxo7* ^−/fl^ mice at 20 weeks (81.3 ± 1.0%; M‐W U = 0.0, *p* = 0.016; Figure 5I and Ta1), and was also present at 46 weeks of age (87.3 ± 3.3%; M‐W U = 6, *p* = 0.02; Figure 5E,F,I and Table 1). A similar decrease was observed in TH^+^ cells of the VTA in *Dat* ^*Cre*^ *Fbxo7* ^−/fl^ mice at 46 weeks (88.84 ± 3.14%; M‐W U = 5, *p* = 0.013; Table 1). These results demonstrate a slow loss of TH^+^ cells in the VM of the *Dat* ^*Cre*^ *Fbxo7* ^−/fl^ mice.

### Coordination, but not locomotion, is affected in conditional *Fbxo7* knockout mice

To determine if reduction in TH^+^ fibre density or loss of DA neurons had behavioural effects, we assessed *Dat* ^*Cre*^ *Fbxo7* ^−/fl^ mice using rotarod and open‐field locomotion tests at 12, 20, and 30 weeks of age. At 12 weeks, there was no significant difference between the animals; however, by 20 weeks, we found a significant reduction in latency to fall on the rotarod, which was also evident at 30 weeks (Figure 5L). In open field tests, we assessed the distance travelled over 10 min, at 20, 30, and 40 weeks of age, and found no significant differences at any age (data not shown). These results show that *Dat* ^*Cre*^ *Fbxo7* ^−/fl^ mice develop a defect in fine motor coordination, but not general locomotion, over time.

### Conditional *Fbxo7* knockout mice have increased levels of RPL23 and upregulate p53‐dependent apoptotic genes

**Figure 6:**
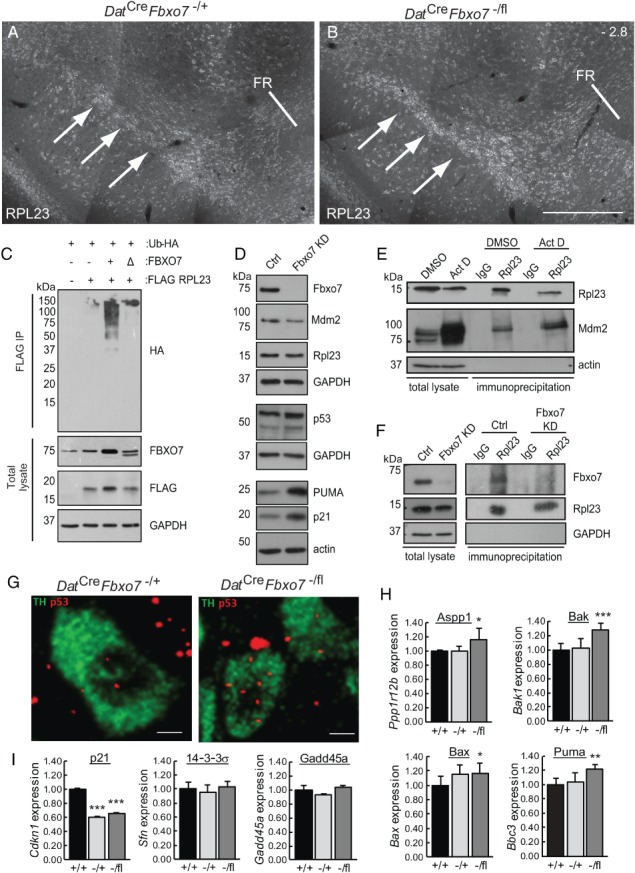
Mutant mice show increased RPL23 and elevated expression of *Trp53* mRNA and p53‐regulated pro‐apoptotic genes. (A, B) Immunofluorescence for RPL23 (arrows) in the SNpc of control (A) and *Dat* ^*Cre*^ *Fbxo7* ^−/fl^ (B) mice at bregma stage −2.8. FR, fasciculus retroflexus (*n* = 3). The scale bar in B represents 200 μm for A, B. (C) *In vivo* ubiquitination assay of RPL23 using HEK293T cells transfected with plasmids expressing FLAG‐RPL23, HA‐tagged ubiquitin, and either untagged WT (+) or ΔF‐box (Δ) FBXO7 constructs. Immunoblots of total lysate prior to anti‐FLAG immunoprecipitation are also shown. (D) Immunoblotting for the expression of various proteins as indicated from cell lysates of SHSY‐5Y cells either constitutively expressing an shRNA targeting *Fbxo7* expression or a control. (E) Co‐immunoprecipitation assays from SHSY‐5Y cells treated for 8.5 h with 5 nm actinomycin D or vehicle (DMSO). Lysates were immunoprecipitated with antibodies to RPL23 and immunoblotted for MDM2. (F) Co‐immunoprecipitation assays from SHSY‐5Y cells with endogenous (Ctrl) or reduced expression of FBXO7. Cells were treated for 4 h with 5 nm actinomycin D and then for a further 5 h with MG132 prior to harvesting. Cell lysates were immunoprecipitated with RPL23 antibodies and immunoblotted with antibodies to Fbxo7. (G) *Th* (green) and *Trp53* (red) transcripts were labelled using branched DNA amplification *in situ* hybridisation. (H, I) RT‐qPCR analysis of p53‐regulated genes isolated from dissected midbrains isolated from *Dat* ^*Cre*^ *Fbxo7* ^+/+^ (+/+), *Dat* ^*Cre*^ *Fbxo7* ^−/+^ (−/+), and *Dat* ^*Cre*^ *Fbxo7* ^−/fl^ (−/fl) mice. Expression was normalised to three reference genes (*Actb*, *Ppia*, *Gapdh*) and expressed relative to WT levels. *p < 0.05, **p < 0.01, ***p < 0.001.

To investigate why FBXO7 loss may cause a reduction in the number of DA neurons in the SNpc and VTA, we investigated pathways highlighted from published screens for FBXO7 substrates 24, 25. Components of the ribosome and proteasome are significantly enriched classes of proteins interacting with SCF^Fbxo7^ ligase 24; moreover, ribosomal proteins were significantly enriched in a ubiquitination protein array by SCF^Fbxo7^ 24. We noted that two ribosomal proteins, RPL11 and RPL23, which act as sensors of cellular stresses, including ribosomal stress, protein misfolding, and nutrient depletion 26, 27, are highly expressed in dopaminergic neurons (Allen Institute for Brain Science, Allen Mouse Brain Atlas; available from: http://mouse.brain-map.org/experiment/show/74819237; last accessed December 2015). To survey RPL expression, VMs were dissected out from *Dat* ^*Cre*^ *Fbxo7* ^−/+^ and *Dat* ^*Cre*^ *Fbxo7* ^−/fl^ mice at 5 weeks of age; whole tissue lysates were separated by SDS‐PAGE, and gel lane slices were subjected to in‐gel digestion (IGD). Peptide fragments were analysed by LC–MS/MS and examined using label free quantitation (Scaffold Proteome software), which identified 2168 unique proteins. Data are available via ProteomeXchange with identifier PXD011666. Within this dataset, only RPL23 was elevated in the *Dat* ^*Cre*^ *Fbxo7* ^−/fl^ VM, whereas 22 other ribosomal proteins were not substantially changed. To verify this, we assayed directly for RPL23 by IHC and found that its expression increased in DA neurons in *Dat* ^*Cre*^ *Fbxo7* ^−/fl^ mice (Figure 6A,B). We first tested whether these proteins interacted in HEK293T and SHSY‐5Y cells by co‐immunoprecipitation assays which were transfected with empty vector or an N‐terminally FLAG‐tagged human FBXO7 expression construct. Endogenous RPL23 was detected in FLAG‐FBXO7, but not in control, immunoprecipitates, indicating that the two proteins interact in human cells (see supplementary material, Figure S1C). This finding raised the possibility that FBXO7 promoted ubiquitination of RPL23. To test this, a HA‐tagged ubiquitin construct was co‐transfected with RPL23 and FBXO7 constructs. In the presence of overexpressed WT, but not the ligase‐dead ΔF‐box mutant, a ladder of poly‐ubiquitinated RPL23 was detected (Figure 6C), demonstrating that FBXO7 promotes the ubiquitination of RPL23. Additionally, we found that expression of WT, but not mutant, FBXO7 led to statistically significant decreased levels of RPL23, and that also treatment with MG132 prevented the reduction in endogenous RPL23 levels seen upon FBXO7 overexpression (see supplementary material, Figure S1D,E). These data indicate that FBXO7 ubiquitinates RPL23, which promotes its degradation by the proteasome.

It is well established that under stress conditions, excess levels of certain free ribosomal subunits directly bind and inhibit MDM2/HDM2, the ubiquitin ligase which promotes p53 degradation, leading to its stabilisation and transactivation of its transcriptional network 26, 27, 28. We tested whether Fbxo7 might also regulate the Rpl23–Mdm2–p53 pathway in neuronal cells. To examine this, we engineered SHSY‐5Y cells with stable expression of an shRNA targeting *Fbxo7* causing a constitutive reduction in its expression. Whole cell lysates were immunoblotted for the levels of Rpl23–Mdm2–p53. As seen in Figure 6D, levels of Mdm2 were decreased, while p53 levels were increased in Fbxo7 KD cells. Moreover, downstream targets of p53 activation, such as p21 and PUMA, were also elevated. Although the steady‐state levels of Rpl23 were unchanged in these cells, immunoprecipitations from cell lysates revealed the interaction between endogenous Rpl23 and Mdm2, which increased upon treatment with actinomycin D to induce ribosomal stress (Figure 6E). Rpl23 also co‐immunoprecipitated with endogenous Fbxo7 in SHSY‐5Y cells when they were treated with actinomycin D and MG132 (Figure 6F). These data indicate that Fbxo7 interacts with Rpl23 to regulate the Rpl23–Mdm2–p53 stress response in neuronal cells.

Since RPL23 was elevated in the substantia nigra of the mutant mice, we tested whether the p53 pathway was activated in these cells. We analysed *Trp53* mRNA expression in the 5‐week‐old mouse brains using *in situ* hybridisation. Samples were also assessed for *Th* mRNA expression. We found low level of *Trp53* expression through the brain sections, and quantified the amount of *Trp53* signal in TH^+^ cells in control and mutant mice. We found a 38% increase in *Trp53* mRNA in *Th* ^+^ cells from *Dat* ^*Cre*^ *Fbxo7* ^−/fl^ mice compared with the control animals (11 ± 4.5 pixels per cell mutant; 8 ± 5 pixels per cell control; Student's *t*‐test, *p* = 0.024) (Figure 6G). Immunoblotting for p53 from whole tissue lysates of dissected VM from 5‐week‐old mice also showed a two‐fold increase in p53 protein expression in *Dat* ^*Cre*^ *Fbxo7* ^−/fl^ mice (see supplementary material, Figure S1F). We also assayed for downstream targets of p53 transcriptional activity by performing RT‐qPCR for p53‐responsive genes on RNA isolated from dissected VM from *Dat* ^*Cre*^ *Fbxo7* ^−/fl^ mice, compared with *Dat* ^*Cre*^ *Fbxo7* ^−/+^ and *Dat* ^*Cre*^ *Fbxo7* ^+/+^ mice. Interestingly, in these post‐mitotic neurons, only p53‐responsive genes regulating apoptosis were significantly upregulated in *Dat* ^*Cre*^ *Fbxo7* ^−/fl^ mice [*Aspp1*, *Bak1*, *Bax*, *Puma* (*Bbc3*); Figure 6H]. Genes associated with cell cycle arrest [p21 (*Cdkn1*), 14‐3‐3σ (*Sfn*), GADD45a (*Gadd45a*); Figure 6I] were unchanged or reduced. These samples were harvested at 5 weeks when there was no obvious cell loss in the VM. Therefore, we predicted that an anti‐apoptotic pathway must be activated to counteract the pro‐apoptotic programme. To test this, RT‐qPCR was performed for Bcl‐2 (*Bcl2*) and Bcl‐xL (*Bcl2l1*) genes. Both transcripts were significantly increased in expression in the mutant samples but not in controls (see supplementary material, Figure S1G). These data suggest that loss of Fbxo7 expression caused an increase in Rpl23 levels, resulting in a pro‐apoptotic p53 transcriptional signature in the midbrains of mutant mice.

Collectively, these data support a model whereby the loss of FBXO7 expression in dopaminergic neurons affects a number of pathways which lead to cell loss in the SNpc, and a locomotor defect – two hallmark features of Parkinson's disease – from 20 weeks of age. Two phenotypes which precede the loss of TH^+^ cells were increased RPL23 expression and decreased cell size and fibre density. Additionally, we observed a robust compensatory mechanism involving the increased expression of TH in the striatum; however, this was not able to rescue the locomotor defects or TH^+^ cell loss in the SNpc and the VTA.

## Discussion

In this study, we investigated the roles that FBXO7 may have in DA neurons. Mice with complete loss of *Fbxo7* in *Dat* ^*Cre*^‐expressing cells developed phenotypes which included some cellular and motor features associated with PD. These phenotypes included reduced TH^+^ fibre density in the striatum, with reduced DA and increased noradrenaline at 6 weeks, followed by TH^+^ cell loss in the SNpc and motor coordination defects by 20 weeks. Innervation of the striatum by fibres originating from the SNpc DA neurons progresses from E16 until approximately 8–9 weeks of age 21. Striosome formation was normal in *Dat* ^*Cre*^ *Fbxo7* ^−/fl^ mice at P0, suggesting that SNpc DA axon guidance was functional. We observed no obvious deviation of TH^+^ fibres from along the medial forebrain bundle. It is possible that neurite outgrowth and/or synaptic signalling are defective in the *Dat* ^*Cre*^ *Fbxo7* ^−/fl^ mice. While our study does not distinguish among these possibilities, the conditional loss of *Fbxo7* post‐innervation might address this and is an area for future research. However, the sustained reduction in DA levels could contribute to the later cellular phenotypes, including reduced TH^+^ cell size in the SNpc and subsequent cell loss. A similar study using *Th* ^*Cre*^ to conditionally delete *Fbxo7* in midbrain DA neurons has been published 25. The authors observed a 50% reduction in DA in the striatum at 2 and 12 months, but no differences in TH^+^ fibre staining or cell loss in *Th* ^*Cre*^ *Fbxo7* ^fl/fl^ mice 25. We speculate that the later onset of *Dat* expression in midbrain DA neurons compared with *Th*, which has an earlier and wider expression pattern, may account for the differences in these models. Experiments using temporally separated Cre lines will be required to address this.

The ectopic presence of TH^+^ cells in the striatum of the *Dat* ^*Cre*^ *Fbxo7* ^−/fl^ mice, though remarkable, is not unique to this study 23, 29. TH^+^ cells occur naturally in the striatum 29, increasing in number following loss of nigrostriatal input in surgically lesioned animals and in transgenic mice with mutations in DA‐associated genes 30, 31, 32, 33, 34. They have also been observed in healthy and Parkinsonian post‐mortem human brains 35, 36. The increased number of TH^+^ cells following denervation of the striatum and administration of l‐dopa has led to speculation that these TH^+^ cells form a compensatory mechanism 22. Previous reports indicated the TH^+^ striatal cells are a type of GABAergic interneuron that expresses TH but not additional enzymes or transporters of dopaminergic neurons, and thus do not release DA 37. It will be interesting to assess whether this is replicated in *FBXO7/PARK15* patients, although no histological reports currently exist.

Our data indicate that FBXO7 negatively regulates the ribosomal sensor RPL23. Many factors can alter ribosome subunit stoichiometry, including fluctuations in cellular metabolism, disruption of rRNA expression, or an imbalance in ribosomal protein production 27. When this occurs, ribosomal protein sensors activate a stress response via a p53 pathway 26, 27. Our data are consistent with a model whereby FBXO7 normally promotes the ubiquitin‐mediated degradation of excess RPL23 subunits. However, in the absence of FBXO7, increased levels of RPL23 titrate away MDM2, leading to increased p53 levels, which we observed in SHSY‐5Y cells and in TH^+^ cells from the midbrain at the mRNA and protein level. Moreover, these 5‐week‐old mutant mice have increased expression of p53‐regulated pro‐apoptotic genes, while p53‐regulated cell cycle genes, such as p21, 14‐3‐3σ or GADD45a, were repressed or unchanged. This is likely due to the upregulation of Aspp1 known to recruit p53 specifically to pro‐apoptotic gene promoters and hinder binding of p53 to the *p21* (*Cdkn1*) promoter 38. Other transactivators, such as Myc and Miz1, may also be causing p53‐mediated repression of p21 39, 40. It is known that p53‐mediated regulation of the ratio of Bax to Bcl‐2 protein levels influences cell fate in response to various cellular stresses. We predicted that there should be an increased anti‐apoptotic signal (upregulation of *Bcl‐2* and *Bcl‐xL* mRNAs) to counteract the pro‐apoptotic signal, which is what we observed. The net effect of these increased *Bax* and *Bcl‐2* mRNAs is that the ratio between these two effectors is unchanged, and this likely accounts for the lack of cell death at 5 weeks of age when these samples were analysed. However, between 6 and 20 weeks, these animals experience significant cell loss in the SNpc and locomotor defects. One possibility is that sustained p53 stabilisation and accumulation over time enable p53's pro‐apoptotic activity directly on mitochondria 41, 42, 43. Another possibility is that these cells have a greater susceptibility to undergo apoptosis, and additional factors, such as proteasomal or oxidative stress, contribute to cell death. Multiple studies indicate that FBXO7 loss can cause defects in proteasomal assembly and activity and impair mitophagy, potentially increasing cell stress 13, 25, 44. The complex phenotypes seen in this mouse argue that multiple pathways are regulated by FBXO7 in the midbrain, which potentially represent avenues of therapeutic intervention during disease progression. FBXO7 models represent valuable tools to study the dynamic molecular processes involved in neurodegeneration.

## Author contributions statement

SRWS, SJR, HL designed and performed experiments; collected, analysed, and interpreted data; and wrote the manuscript. RAB and JWD supervised experiments, and SAR, RH, BM, PAR, and JX performed experiments. All authors had final approval of the submitted manuscript.

**SUPPLEMENTARY MATERIAL ONLINE**

**Supplementary materials and methods**

**Supplementary figure legend**

**Figure S1.** Mice lacking Fbxo7 in dopaminergic neurons have increased Rpl23 and increased p53 signalling

**Table S1.** List of primers used in this study (mentioned in supplementary material, Supplementary materials and methods)

## Supporting information


**Supplementary materials and methods**

**Supplementary figure legend**
**Figure S1**. Mice lacking Fbxo7 in dopaminergic neurons have increased Rpl23 and increased p53 signalling

**Table S1.** List of primers used in this study.

## References

[1] de Lau LM , Breteler MM . Epidemiology of Parkinson's disease. Lancet Neurol 2006; 5: 525–535. 16713924 10.1016/S1474-4422(06)70471-9

[2] Lill CM . Genetics of Parkinson's disease. Mol Cell Probes 2016; 30: 386–396. 27818248 10.1016/j.mcp.2016.11.001

[3] Blesa J , Przedborski S . Parkinson's disease: animal models and dopaminergic cell vulnerability. Front Neuroanat 2014; 8: 155. 25565980 10.3389/fnana.2014.00155 PMC4266040

[4] Nelson DE , Randle SJ , Laman H . Beyond ubiquitination: the atypical functions of Fbxo7 and other F‐box proteins. Open Biol 2013; 3: 130131. 24107298 10.1098/rsob.130131 PMC3814724

[5] Shojaee S , Sina F , Banihosseini SS , *et al* Genome‐wide linkage analysis of a Parkinsonian‐pyramidal syndrome pedigree by 500 K SNP arrays. Am J Hum Genet 2008; 82: 1375–1384. 18513678 10.1016/j.ajhg.2008.05.005 PMC2427312

[6] Bartonikova T , Mensikova K , Mikulicova L , *et al* Familial atypical parkinsonism with rare variant in *VPS35* and *FBXO7* genes: a case report. Medicine (Baltimore) 2016; 95: e5398. 27861377 10.1097/MD.0000000000005398 PMC5120934

[7] Lohmann E , Coquel AS , Honore A , *et al* A new F‐box protein 7 gene mutation causing typical Parkinson's disease. Mov Disord 2015; 30: 1130–1133. 26010069 10.1002/mds.26266

[8] Yalcin‐Cakmakli G , Olgiati S , Quadri M , *et al* A new Turkish family with homozygous *FBXO7* truncating mutation and juvenile atypical parkinsonism. Parkinsonism Relat Disord 2014; 20: 1248–1252. 25085748 10.1016/j.parkreldis.2014.06.024

[9] Chen CM , Chen IC , Huang YC , *et al* *FBXO7* Y52C polymorphism as a potential protective factor in Parkinson's disease. PLoS One 2014; 9: e101392. 25029497 10.1371/journal.pone.0101392 PMC4100735

[10] Gunduz A , Eken AG , Bilgic B , *et al* *FBXO7–R498X* mutation: phenotypic variability from chorea to early onset parkinsonism within a family. Parkinsonism Relat Disord 2014; 20: 1253–1256. 25169713 10.1016/j.parkreldis.2014.07.016

[11] Paisan‐Ruiz C , Guevara R , Federoff M , *et al* Early‐onset L‐dopa‐responsive parkinsonism with pyramidal signs due to *ATP13A2*, *PLA2G6*, *FBXO7* and *spatacsin* mutations. Mov Disord 2010; 25: 1791–1800. 20669327 10.1002/mds.23221 PMC6005705

[12] Di Fonzo A , Dekker MC , Montagna P , *et al* *FBXO7* mutations cause autosomal recessive, early‐onset parkinsonian‐pyramidal syndrome. Neurology 2009; 72: 240–245. 19038853 10.1212/01.wnl.0000338144.10967.2b

[13] Burchell VS , Nelson DE , Sanchez‐Martinez A , *et al* The Parkinson's disease‐linked proteins Fbxo7 and Parkin interact to mediate mitophagy. Nat Neurosci 2013; 16: 1257–1265. 23933751 10.1038/nn.3489 PMC3827746

[14] Rodriguez CI , Buchholz F , Galloway J , *et al* High‐efficiency deleter mice show that FLPe is an alternative to Cre‐*loxP* . Nat Genet 2000; 25: 139–140. 10835623 10.1038/75973

[15] Lewandoski M , Wassarman KM , Martin GR . *Zp3–cre*, a transgenic mouse line for the activation or inactivation of *loxP*‐flanked target genes specifically in the female germ line. Curr Biol 1997; 7: 148–151. 9016703 10.1016/s0960-9822(06)00059-5

[16] Zhuang X , Masson J , Gingrich JA , *et al* Targeted gene expression in dopamine and serotonin neurons of the mouse brain. J Neurosci Methods 2005; 143: 27–32. 15763133 10.1016/j.jneumeth.2004.09.020

[17] Stott SR , Hayat S , Carnwath T , *et al* CD24 expression does not affect dopamine neuronal survival in a mouse model of Parkinson's disease. PLoS One 2017; 12: e0171748. 28182766 10.1371/journal.pone.0171748 PMC5300212

[18] Randle SJ , Nelson DE , Patel SP , *et al* Defective erythropoiesis in a mouse model of reduced Fbxo7 expression due to decreased p27 expression. J Pathol 2015; 237: 263–272. 26095538 10.1002/path.4571 PMC4583784

[19] Meziane el K , Randle SJ , Nelson DE , *et al* Knockdown of Fbxo7 reveals its regulatory role in proliferation and differentiation of haematopoietic precursor cells. J Cell Sci 2011; 124: 2175–2186. 21652635 10.1242/jcs.080465

[20] Stott SR , Metzakopian E , Lin W , *et al* Foxa1 and Foxa2 are required for the maintenance of dopaminergic properties in ventral midbrain neurons at late embryonic stages. J Neurosci 2013; 33: 8022–8034. 23637192 10.1523/JNEUROSCI.4774-12.2013 PMC6618950

[21] Keller HH , Bartholini G , Pletscher A . Spontaneous and drug‐induced changes of cerebral dopamine turnover during postnatal development of rats. Brain Res 1973; 64: 371–378. 4781347 10.1016/0006-8993(73)90190-x

[22] Bezard E , Gross CE . Compensatory mechanisms in experimental and human Parkinsonism: towards a dynamic approach. Prog Neurobiol 1998; 55: 93–116. 9618745 10.1016/s0301-0082(98)00006-9

[23] Nakahara T , Yamamoto T , Endo K , *et al* Neuronal ectopic expression of tyrosine hydroxylase in the mouse striatum by combined administration of 1‐methyl‐4‐phenyl‐1,2,3,6‐tetrahydropyridine and 3‐nitropropionic acid. Neuroscience 2001; 108: 601–610. 11738497 10.1016/s0306-4522(01)00441-9

[24] Teixeira FR , Randle SJ , Patel SP , *et al* Gsk3β and Tomm20 are substrates of the SCFFbxo7/PARK15 ubiquitin ligase associated with Parkinson's disease. Biochem J 2016; 473: 3563–3580. 27503909 10.1042/BCJ20160387 PMC5260939

[25] Vingill S , Brockelt D , Lancelin C , *et al* Loss of FBXO7 (PARK15) results in reduced proteasome activity and models a parkinsonism‐like phenotype in mice. EMBO J 2016; 35: 2008–2025. 27497298 10.15252/embj.201593585 PMC5282834

[26] Sung MK , Reitsma JM , Sweredoski MJ , *et al* Ribosomal proteins produced in excess are degraded by the ubiquitin–proteasome system. Mol Biol Cell 2016; 27: 2642–2652. 27385339 10.1091/mbc.E16-05-0290 PMC5007085

[27] Zhou X , Liao WJ , Liao JM , *et al* Ribosomal proteins: functions beyond the ribosome. J Mol Cell Biol 2015; 7: 92–104. 25735597 10.1093/jmcb/mjv014 PMC4481666

[28] Dai MS , Zeng SX , Jin Y , *et al* Ribosomal protein L23 activates p53 by inhibiting MDM2 function in response to ribosomal perturbation but not to translation inhibition. Mol Cell Biol 2004; 24: 7654–7668. 15314173 10.1128/MCB.24.17.7654-7668.2004 PMC506971

[29] Tashiro Y , Sugimoto T , Hattori T , *et al* Tyrosine hydroxylase‐like immunoreactive neurons in the striatum of the rat. Neurosci Lett 1989; 97: 6–10. 2563908 10.1016/0304-3940(89)90130-4

[30] Jaber M , Dumartin B , Sagne C , *et al* Differential regulation of tyrosine hydroxylase in the basal ganglia of mice lacking the dopamine transporter. Eur J Neurosci 1999; 11: 3499–3511. 10564358 10.1046/j.1460-9568.1999.00764.x

[31] Huot P , Parent A . Dopaminergic neurons intrinsic to the striatum. J Neurochem 2007; 101: 1441–1447. 17286588 10.1111/j.1471-4159.2006.04430.x

[32] Kadkhodaei B , Ito T , Joodmardi E , *et al* Nurr1 is required for maintenance of maturing and adult midbrain dopamine neurons. J Neurosci 2009; 29: 15923–15932. 20016108 10.1523/JNEUROSCI.3910-09.2009 PMC6666174

[33] Espadas I , Darmopil S , Vergaño‐Vera E , *et al* L‐DOPA‐induced increase in TH‐immunoreactive striatal neurons in parkinsonian mice: insights into regulation and function. Neurobiol Dis 2012; 48: 271–281. 22820144 10.1016/j.nbd.2012.07.012

[34] Doucet‐Beaupre H , Gilbert C , Profes MS , *et al* Lmx1a and Lmx1b regulate mitochondrial functions and survival of adult midbrain dopaminergic neurons. Proc Natl Acad Sci U S A 2016; 113: E4387–E4396. 27407143 10.1073/pnas.1520387113 PMC4968767

[35] Porritt MJ , Batchelor PE , Hughes AJ , *et al* New dopaminergic neurons in Parkinson's disease striatum. Lancet 2000; 356: 44–45. 10892768 10.1016/S0140-6736(00)02437-5

[36] Cossette M , Lecomte F , Parent A . Morphology and distribution of dopaminergic neurons intrinsic to the human striatum. J Chem Neuroanat 2005; 29: 1–11. 15589697 10.1016/j.jchemneu.2004.08.007

[37] Xenias HS , Ibanez‐Sandoval O , Koos T , *et al* Are striatal tyrosine hydroxylase interneurons dopaminergic? J Neurosci 2015; 35: 6584–6599. 25904808 10.1523/JNEUROSCI.0195-15.2015 PMC4405564

[38] Samuels‐Lev Y , O'Connor DJ , Bergamaschi D , *et al* ASPP proteins specifically stimulate the apoptotic function of p53. Mol Cell 2001; 8: 781–794. 11684014 10.1016/s1097-2765(01)00367-7

[39] Herold S , Wanzel M , Beuger V , *et al* Negative regulation of the mammalian UV response by Myc through association with Miz‐1. Mol Cell 2002; 10: 509–521. 12408820 10.1016/s1097-2765(02)00633-0

[40] Seoane J , Le HV , Massague J . Myc suppression of the *p21* ^*Cip1*^ Cdk inhibitor influences the outcome of the p53 response to DNA damage. Nature 2002; 419: 729–734. 12384701 10.1038/nature01119

[41] Culmsee C , Mattson MP . p53 in neuronal apoptosis. Biochem Biophys Res Commun 2005; 331: 761–777. 15865932 10.1016/j.bbrc.2005.03.149

[42] Perier C , Bove J , Wu DC , *et al* Two molecular pathways initiate mitochondria‐dependent dopaminergic neurodegeneration in experimental Parkinson's disease. Proc Natl Acad Sci U S A 2007; 104: 8161–8166. 17483459 10.1073/pnas.0609874104 PMC1876588

[43] Wang DB , Kinoshita C , Kinoshita Y , *et al* p53 and mitochondrial function in neurons. Biochim Biophys Acta 2014; 1842: 1186–1197. 24412988 10.1016/j.bbadis.2013.12.015 PMC4074561

[44] Zhou ZD , Xie SP , Sathiyamoorthy S , *et al* F‐box protein 7 mutations promote protein aggregation in mitochondria and inhibit mitophagy. Hum Mol Genet 2015; 24: 6314–6330. 26310625 10.1093/hmg/ddv340

