# Supplementary material for: Loss of FBXO7 results in a Parkinson's‐like dopaminergic degeneration via an RPL23–MDM2–TP53 pathway
Source: J Pathol. 2019 Aug 6;249(2):241–54. doi: 10.1002/path.5312 (PMC6790581; doi:10.1002/path.5312)
Supplement: Supplementary file 1 — Supplementary materials and methods [file PATH-249-241-s001.docx]

**Loss of FBXO7 results in a Parkinson’s-like dopaminergic degeneration via an RPL23–MDM2–TP53 pathway**

Stott SRW *et al*. *J Pathol* DOI: 10.1002/path.5312

**Supplementary materials and methods**

*Mice*

All experiments in mice were performed in accordance with the UK Animals (Scientific Procedures) Act 1986 and ARRIVE guidelines. Animal licences were approved by the Home Office and the University of Cambridge’s Animal Welfare & Ethical Review Body Standing Committee. Experiments were performed under Home Office licences PPL 80/2474, 70/9001, 80/2366, and 70/8411.

*Behavioural testing*

Mice were tested using rotarod and open field locomotion assessments. For the rotarod (LE8200; Panlab**/Harvard Apparatus, Cambridge, UK**), mice were trained for 3 days (with a 3-day rest period in between sessions). Day 2 involved repeating the 16 rpm test for 1 min for the first two trials, following which it was shifted to 24 rpm for the last two trials (20 min apart); day 3 training required 1 min at 24 rpm for four trials (20 min apart). Testing was conducted over 2 days (with a 3-day rest period in between), involving four recorded trials: two trials of 24 rpm, followed by two trials of 16 rpm. The open field activity measure was conducted over 2 days (with a 3-day rest period in between) and involved a 30-min habituation period on the first day and a 10-min recording session on the second day. The testing arena was 70 cm × 55 cm × 22 cm, with a Logitech-c170 webcam recording movement from above the enclosure. Footage of test sessions was analysed using Icy ‘Mice Profiler Tracking’ software, Version Icy.1.5.4.2 (http://icy.bioimageanalysis.org/; downloaded 9 July 2014; last accessed 29 December 2014).

*Tissue processing*

Tissues were taken at indicated time points. For HPLC and immunoblotting analysis, mice were culled by cervical dislocation and brains were snap-frozen on dry ice. The brain regions collected included the olfactory bulb, prefrontal cortex, striatum, and ventral midbrains, which were dissected from cryostat-cut 30-μm-thick coronal sections. For all other experiments, mice were euthanized with a 0.5 ml intraperitoneal injection of Euthatal (pentobarbitone sodium, 200 mg/ml; Merial Animal Health Ltd, Boehringer Ingelheim Animal Health, Bracknell, Berkshire, UK) and perfused transcardially with 0.9% saline followed by ice-cold 4% paraformaldehyde (0.1 m phosphate buffer, pH 7.4). Brains were removed and post-fixed in 4% PFA overnight, and then placed in 30% sucrose.

*Immunohistochemistry*

Perfused brains were sectioned coronally at 35-µm intervals using a sledge microtome [Leica SM 2400, Leitz 1400; Leica Microsystems (UK) Ltd, Milton Keynes, Buckinghamshire, UK]. Sections were collected in six series and washed in 0.1 m phosphate buffer saline (PBS) before being quenched (3% hydrogen peroxide–10% methanol for 30 min), washed in PBS, and incubated in 5% serum–0.05% Triton X-100. The antibodies used were rabbit anti-CRE (Cat No Poly9080; 1:100; BioLegend, London, UK), rat anti-dopamine transporter (DAT) (Cat No MAB369; 1:200; Chemicon International, Temecula, CA, USA), rabbit anti-DARPP32 (Cat No 2306; 1:200; Cell Signaling Technology, London, UK), mouse anti-NeuN (Cat No MAB377; 1:100; Merck Millipore, Watford, Hertfordshire, UK), rabbit anti-RPL23 (Cat No HPA003373; 1:200; Atlas Antibodies, Bromma, Sweden), rabbit anti-tyrosine hydroxylase (TH) (Cat No P4010-1; 1:1000; Pel-Freez Biologicals, Rogers, AR, USA), mouse anti-TH (Cat No MAB318; 1:500, Merck Millipore), and sheep anti-TH (AB1542; 1:1000; Merck Millipore). Signals were visualised by immunofluorescence (Alexa Fluor^®^ secondary antibodies: donkey anti-mouse Alexa Fluor^®^ 488 (Cat No A-21202), donkey anti-rabbit Alexa Fluor^®^ 488 (Cat No A-21206), donkey anti-mouse Alexa Fluor^®^ 555 (Cat No A-31570), donkey anti-rabbit Alexa Fluor^®^ 555 (Cat No A-31572), donkey anti-sheep Alexa Fluor^®^ 568 (Cat No A-21099), goat anti-rat Alexa Fluor^®^ 680 (Cat No A-21096) (all at 1:500; Thermo Fisher Scientific, Loughborough, UK), or diaminobenzidine (DAB; Sigma-Aldrich, Gillingham, Dorset, UK). Multiple PBS washes were followed by 1 h incubation of the appropriate secondaries in blocking solution, followed by multiple PBS washes. Immunohistochemical staining was visualised using the VECTASTAIN^®^ Avidin-Biotin Complex (ABC) Staining Kit (Vector Labs). Sections were washed multiple times in PBS before being incubated for 45 min in ABC solution, followed by multiple PBS washes and incubation in diaminobenzidine (DAB; Sigma-Aldrich).

*Stereological analysis*

Randomised estimations of the total number of TH^+^ cells in the ventral midbrains of the mice were performed using a standard stereological method (Olympus CAST Grid System). Substantia nigra (SN) incorporated both the SN pars compacta (SNpc) and the SN pars reticulata (SNpr), and was defined by drawing a vertical line through the most medial tip of the cerebral peduncle which represented the medial border of the SN. The dorsal border of the SN extended dorsolaterally along the upper limits of the cells populating the SNpc, and by so doing, encompassed all of the TH^+^ neurons of the SNpc and the SN pars lateralis. The medial lemniscus provided an additional dividing line between the SN and the VTA on sections where it was available. The ventral perimeter began at the extremity of the pars lateralis and followed the dorsal border of the cerebral peduncle ventrally until it met the medial vertical line, thereby including the TH^+^ cells in the SNpr. For the estimations of the VTA, the dorsal limits of the SN represented the ventral border of the rostral regions of the VTA. TH^+^ cell bodies lining the posterior hypothalamic nucleus were considered the medial limits, while the fasciculus retroflexus was the dorsal border. The diagonally-oriented lateral border of the VTA was defined by the TH^+^ cells lining the midbrain reticular nucleus. For more caudally located regions of the VTA, the border of the midbrain reticular nucleus was considered the lateral border, while the medial border of the VTA gradually became the midline of the ventral midbrain. The intersection of the lateral and medial borders was the most dorsal region counted, and the medial lemniscus represented the ventral border. These rules also applied to the most caudal sections of the VTA. A total of five sections of SN and VTA were counted per brain, from between −2.5 and −3.9 relative to the bregma.

*Optical density analysis*

To determine the fibre density in the striatum and SNpr, the mean optical intensity was measured from the TH-positive stained sections. All DAB-stained slides were imaged using the Scanscope XT slide scanner [Aperio, Leica Microsystems (UK) Ltd] at a resolution of 0.5 μm per pixel, using a ×20 objective. Images for subsequent analysis were captured using Aperio ImageScope [Leica Microsystems (UK) Ltd; <https://www.leicabiosystems.com/digital-pathology/manage/aperio-imagescope/>]. The areas of interest were the striatum, the nucleus accumbens, and the SNpr. For the striatum, the measurements were made on 12 coronal sections, delineated as being between +1.7 and −1.4 relative to the bregma. The lining of the ventricular wall and external capsule represented the medial and dorsal/lateral borders of the defined area of interest in the striatum. The ventral limit of the striatum was a diagonal line passing above the anterior commissure, between the external capsule and the ventricular wall. For more posterior sections, the lateral aspect of the globus pallidus was used as the medial border of the striatum, and a horizontal line was made between the external capsule and the globus pallidus to outline the ventral border. Density measurements for the nucleus accumbens were made across four coronal sections, approximately +1.7 to +1.0, relative to the bregma. A diagonal line passing down through the anterior commissure, between the external capsule and the lowest portion of the ventricular wall, was used as the dorsal border, and the lowest limits of the olfactory tubercle defined the ventral perimeter of the area that we defined as nucleus accumbens. TH staining along the lateral stripe of the striatum represented the lateral wall, and TH^+^ shell of the nucleus accumbens was used as the medial limits. Fibre density analysis on the TH^+^ fibres of the SNpr was conducted across two coronal sections per brain (320 µm apart). The analysed area was defined as the rostral SNpc, where only TH^+^ fibres are present in the SNpr and no TH^+^ cell bodies (from approximately −2.7 to −3.2 according to the bregma). For TH staining, non-specific background was determined by readings made from the TH^−^ corpus callosum and normalised to the white light background surrounding the section on the glass slide. All fibre density analysis was carried out using ImageJ (National Institutes of Health, Bethesda, MD, USA; <https://imagej.nih.gov/ij/>; version 1.46r; downloaded in 2012).

*Cell body size measurements*

Fluorescent images of TH^+^ neurons were captured randomly using a 40× lens on a stereological microscope (Olympus CAST Grid System) and analysed using ImageJ. Across three coronal planes of the midbrain (from approximately −2.7 to −3.4 according to the bregma), three images were captured from the SNpc and three images from the VTA. The area of the soma of SNpc TH^+^ neurons was measured (*N* = 3 of each genotype; at least 200 cells per brain were measured).

*Neurotransmitter measurements*

DA and norepinephrine were measured using reversed-phase high-performance liquid chromatography (HPLC) and electrochemical detection. Samples were homogenised in 0.2 m perchloric acid and centrifuged at 6000 rpm for 10 min at 4°C. Then 25 µl aliquots of supernatant were injected onto a C18 ODS 3 mm column (Hypersil Elite, Phenomenex, Macclesfield, UK) with a mobile phase consisting of citric acid (31.9 g/l), sodium acetate (2 g/l), octanesulfonic acid (460 mg/l), EDTA (30 mg/l), and 15% methanol (pH 3.6). DA and norepinephrine were detected using an ESA Coulochem II detector (ESA Biosciences Inc, Chelmsford, MA, USA) with electrode 1 held at −200 mV and electrode 2 held at +250 mV. Chromatograms were acquired and analysed using Chromeleon software (Dionex Corporation, Sunnyvale, CA, USA).

*Cell culture and transfection*

HEK293T cells were cultured and transfected with empty pcDNA3-Flag vector, or pcDNA3 vectors containing Flag-FBXO7 or Flag-FBXO7-ΔFBOX. Cells were subjected to 6 h of 10 µm MG132 treatment where indicated.

*Lysis, immunoprecipitation, and immunoblotting*

Frozen brain regions were lysed (10 µl per mg tissue) in RIPA buffer [50 mm Tris (pH 7.5), 150 mm NaCl, 0.5% sodium deoxycholate, 1% NP40, 0.1% SDS] with protease inhibitor cocktail and 1 mm PMSF, as well as phosphatase inhibitors (10 mm NaF and 1 mm Na_3_VO_4_), and samples were homogenised using a Dounce homogeniser. Lysates were incubated on ice for 30 min and then centrifuged at 16 100 × *g* for 10 min at 4°C. Protein concentration was determined using a BCA assay (Cat No 23227; Thermo Fisher Scientific), and 100 µg of total protein was subjected to immunoblotting. For cell lysates, cells were lysed as above and 50 µg of the lysate was analysed by immunoblotting. For immunoprecipitation assays, cell lysates were incubated with anti-Flag M2 agarose (Cat No A2220; Sigma-Aldrich) for 4 h at 4°C with rotation; the beads washed four times with ice-cold RIPA buffer; and proteins eluted using Laemmli buffer before analysis by immunoblotting. The following antibodies were used: rabbit anti-RPL23 (Cat No HPA003373; 1:200; Atlas Antibodies, Bromma, Sweden; or Cat No 305-009A; 1:2500; Bethyl Laboratories, Inc, Heidelberg, Germany), mouse anti-Mdm2 (Cat No sc-965; 1:1000; Santa Cruz Biotechnology, Dallas, TX, USA), rabbit anti-p53 (Cat No 9282S; 1:1000; Cell Signaling Technology), mouse anti-PUMA (Cat No sc-377015; 1:500; Santa Cruz Biotechnology), rabbit anti-p21 (Cat No sc-397; 1:1000; Santa Cruz Biotechnology), rabbit anti-actin (Cat No A2066; 1:5000; Sigma-Aldrich), rabbit anti-GAPDH (Cat No G9545; 1:5000; Sigma-Aldrich), and mouse anti-FBXO7 (Cat No sc-271763; 1:250; Santa Cruz Biotechnology). Donkey anti-rabbit IgG (sc-2313; 1:10 000; Santa Cruz Biotechnology) and goat anti-mouse IgG (Cat No sc-2055; 1:10 000; Santa Cruz Biotechnology), conjugated to horseradish peroxidase, were used to visualise bands. Protein quantification was performed using densitometry analysis and ImageJ software. Protein levels were normalised to loading control (actin or GAPDH) and expressed relative to WT or vector control levels.

*Isolation of RNA and RT-qPCR*

Ventral midbrain regions (~10 mg) were micro-dissected from cryostat sections. Tissue was homogenised in 350 µl of RLT buffer with β-mercaptoethanol and RNA was isolated using an RNeasy Plus kit (Qiagen, Manchester, UK) following the manufacturer’s recommendations. One microgram of total RNA was converted to cDNA using Quantitect Reverse Transcriptase (Qiagen) and then diluted 1:10 for subsequent qPCR analysis using SYBR Green JumpStart Taq (Sigma-Aldrich) on a CFX Connect Real-Time PCR machine (Bio-Rad, Watford, UK). Primer sequences are listed in the supplementary material, Table S1, for assessment of the transcript levels for *ActB*, *Aspp1*, *Bak1*, *Bax*, *Bbc3*, *Bcl-2*, *Bcl-2L1*, *Cdkn1a*, *Cre*, *Fbxo7*, *Gadd45a*, *Gapdh*, *Ppiai*, and *Sfn.* Conditions for qPCR reactions were as follows: 95°C for 5 min; then 45 cycles of 95°C for 30 s, 60°C for 30 s, 72°C for 30 s, followed by melt curve analysis to confirm that a single PCR product had been made. Relative gene expression was determined using the relative standard curve method; data were normalised to three reference genes (*Ppai*, *Gapdh*, *Actb*) and expressed relative to WT levels.

In situ *hybridisation imaging and analysis*

*In situ* hybridisation was performed on sections of formalin-fixed, paraffin-embedded (FFPE) brain from 5-week-old mice. Simultaneous fluorescence detection of mouse tyrosine hydroxylase (*Th*) and mouse p53 mRNA (*Trp53*) was accomplished using an Advanced Cell Diagnostics (ACD) RNAscope^®^ 2.5 LS Multiplex Reagent Kit (Cat No 322800), RNAscope^®^ 2.5 LS Probe-Mm Th (Cat No 317628), and RNAscope^®^ 2.5 LS Probe-Mm Trp53-C2 (Cat No 402338-C2) (ACD, Hayward, CA, USA), while single channel colourimetric detection of *Fbxo7* was performed using RNAscope^®^ LS 2.5 Probe-Mm-Fbxo7 (Cat No 457698). In brief, sections were cut at 4 µm thickness and baked for 1 h at 60°C before loading onto a Bond RX instrument [Leica Microsystems (UK) Ltd]. Slides were deparaffinised and rehydrated on-board before pretreatments using Epitope Retrieval Solution 2 [Cat No AR9640; Leica Microsystems (UK) Ltd] at 88°C for 10 min, and ACD Enzyme from the Duplex Reagent kit at 40°C for 10 min. Probe hybridisation and signal amplification were performed according to the manufacturer’s instructions. Fluorescence detection of *Th* and *Tp53* was performed on the Bond Rx using TSA plus Cyanine 5 (Cat No NEL745001KT; Perkin Elmer, Llantrisant, UK) and TSA plus Cyanine 3 (Cat No NEL744001KT; Perkin Elmer), respectively, at a dilution of 1:1000 in ACD TSA diluent according to the ACD protocol. Slides were then removed from the Bond Rx and washed briefly in ultrapure water before being mounted in Prolong Diamond (Cat No P36961; Thermo Fisher Scientific). Fluorescent slides were imaged on a Zeiss LSM 700 confocal microscope (Carl Zeiss Ltd, Cambridge, UK). Z stacks of the VM region were acquired and a maximum projection was performed for each channel. Cells double-positive for *Th* and *Trp53* were identified and quantified. The number of *Trp53* RNA transcripts was based on the fluorescence signal counts from 57 randomly selected cells from control and mutant sample Z-stack projection images. For each spot, every pixel was considered as a positive signal.

*Statistical analysis*

Statistical analysis was performed using statistical software (GraphPad Prism software, version 5.0; GraphPad Inc, San Diego, CA, USA). All comparisons between groups of mice were performed using two-way ANOVA, two-tailed Student’s *t*-test or Mann–Whitney *U*-test (M-W U tests were used for the stereological figures in Table 1). The statistical test and the number of animals used for each test are indicated in the Results section. For western blots, a Kruskal–Wallis test, with Dunn’s multiple comparisons *post hoc* test, was performed. Differences were considered significant when *P* values were less than 0.05 (**p* < 0.05; ***p* < 0.005; ****p* < 0.0005 – unless otherwise indicated). Percentages are presented with the standard error of the mean (SEM) in both the text and the figures.
