# Supplementary material for: Loss of FBXO7 results in a Parkinson's‐like dopaminergic degeneration via an RPL23–MDM2–TP53 pathway
Source: J Pathol. 2019 Aug 6;249(2):241–54. doi: 10.1002/path.5312 (PMC6790581; doi:10.1002/path.5312)
Supplement: Supplementary file 2 — Supplementary figure legend Figure S1. Mice lacking Fbxo7 in dopaminergic neurons have increased Rpl23 and increased p53 signalling [file PATH-249-241-s002.zip › path_5312_SuppFigLegs.docx]

**Loss of FBXO7 results in a Parkinson’s-like dopaminergic degeneration via an RPL23-MDM2–TP53 pathway**

Stott SRW *et al*. *J Pathol* DOI: 10.1002/path.5312

**Figure S1. Mice lacking Fbxo7 in dopaminergic neurons have increased Rpl23 and increased p53 signalling.**

(A) Photograph of WT and *Fbxo7*^−/−^ littermates at P8. (B) TH and CRE staining on sections of brain from the *Dat^Cre^ Fbxo7*^−/+^ mouse at the level of the SNpc. ****p* < 0.001. Scale bars: 1 cm in A and 400 µm in B. (C) Anti-FLAG antibody immunoprecipitations (FLAG IP) from HEK293T (left) and SHSY-5Y (right) cell lysates transfected with FLAG control or FLAG-FBXO7, showing interaction of endogenous RPL23 with FBXO7. Immunoblots of total lysate prior to immunoprecipitation are also shown. (D) Immunoblot of endogenous RPL23 expression in HEK293 cells transfected with FLAG control (vector), FLAG-FBXO7 (FBXO7) or the mutant FLAG-FBXO7-ΔF-box (ΔF-box) constructs. (E) Quantification of RPL23 expression in cells transfected as in D. (F) Immunoblotting for p53 of cell lysates from dissected midbrains from 5-week-old *Dat*^+/+^ *Fbxo7*^+/fl^ mice (Ctrl) and *Dat^Cre^* *Fbxo7*^−/fl^ (mutant) mice. (G) RT-qPCR analysis of p53-regulated genes isolated from dissected midbrains isolated from *Dat^Cre^ Fbxo7*^+/+^ (+/+), *Dat^Cre^ Fbxo7*^−/+^ (−/+), and *Dat^Cre^ Fbxo7*^−/fl^ (−/fl) mice. Expression was normalised to three reference genes (*Actb*, *Ppia*, *Gapdh*) and is expressed relative to WT levels.
