# Supplementary figures and images for: Loss of FBXO7 results in a Parkinson's‐like dopaminergic degeneration via an RPL23–MDM2–TP53 pathway
Source: J Pathol. 2019 Aug 6;249(2):241–54. doi: 10.1002/path.5312 (PMC6790581; doi:10.1002/path.5312)

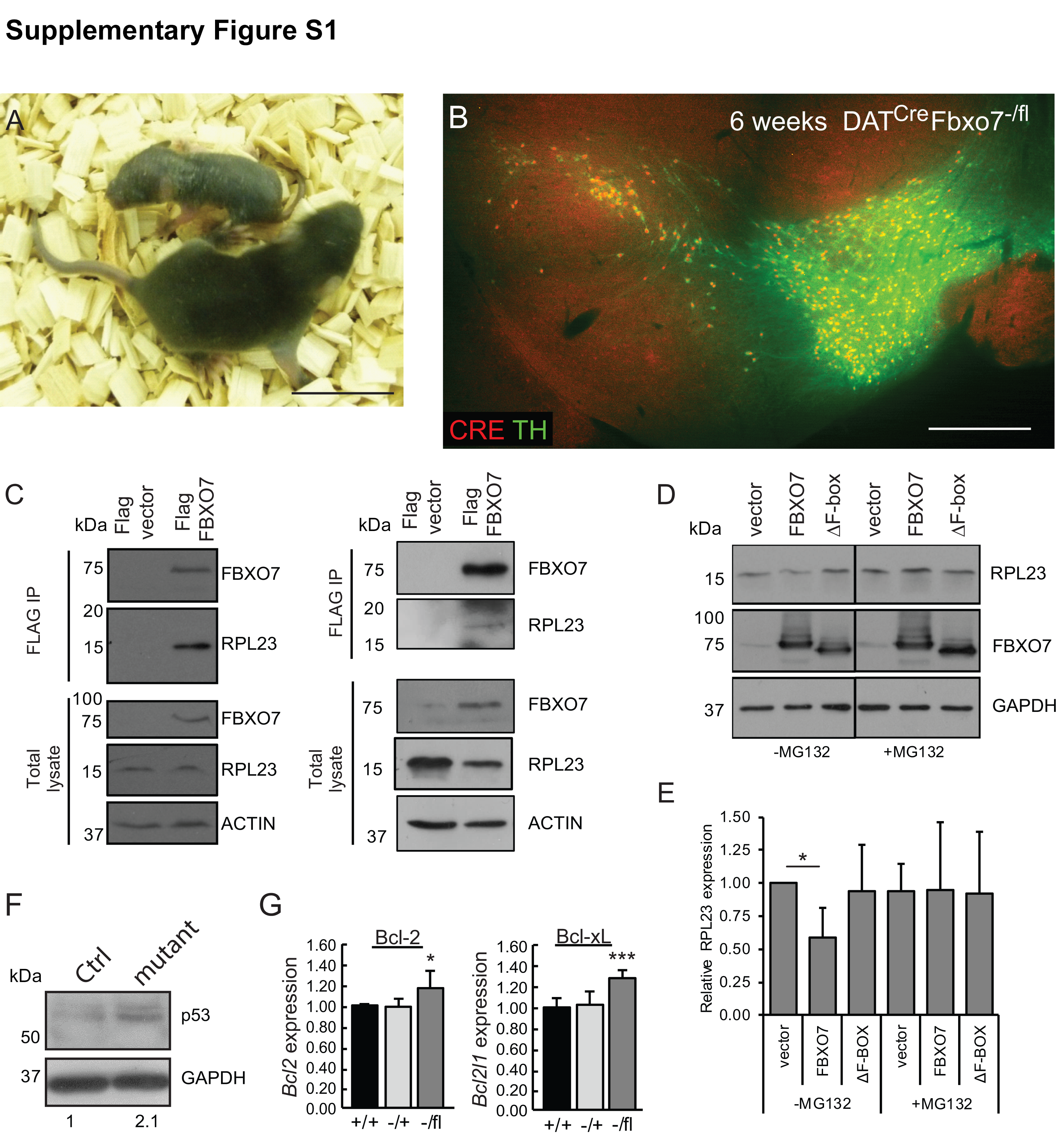

Supplement: Supplementary file 2 — Supplementary figure legend Figure S1. Mice lacking Fbxo7 in dopaminergic neurons have increased Rpl23 and increased p53 signalling [file PATH-249-241-s002.zip › path_5312_Supp_FigS1.tif]
