# Supplementary material for: Loss of FBXO7 results in a Parkinson's‐like dopaminergic degeneration via an RPL23–MDM2–TP53 pathway
Source: J Pathol. 2019 Aug 6;249(2):241–54. doi: 10.1002/path.5312 (PMC6790581; doi:10.1002/path.5312)
Supplement: Supplementary file 3 — Table S1. List of primers used in this study. [file PATH-249-241-s003.docx]

**Loss of FBXO7 results in a Parkinson’s-like dopaminergic degeneration via an RPL23–MDM2–TP53 pathway**

Stott SRW *et al*. *J Pathol* DOI: 10.1002/path.5312

**Table S1.** List of primers used in this study

| Mm ActB F | GGCACCACACCTTCTACAATG |
| --- | --- |
| Mm ActB R | GGGGTGTTGAAGGTCTCAAAC |
| Mm Aspp1 F | CGCAAAGATGAAAACGAGAC |
| Mm Aspp1 R | CACCTTGCTCTTGTTCAAGG |
| Mm Bak1 F | ATATTAACCGGCGCTACGAC |
| Mm Bak1 R | AGGCGATCTTGGTGAAGAGT |
| Mm Bax F | AAGCTGAGCGAGTGTCTCCGGCG |
| Mm Bax R | GCCACAAAGATGGTCACTGTCTGCC |
| Mm Bbc3 F | ATGGCGGACGACCTCAAC |
| Mm Bbc3 R | AGTCCCATGAAGAGATTGTACATGAC |
| Mm Bcl-2L1F | TGGAGTAAACTGGGGGTCGCATCG |
| Mm Bcl-2L1 R | AGCCACCGTCATGCCCGTCAGG |
| Mm Bcl-2 F | CTCGTCGCTACCGTCGTGACTTCG |
| Mm Bcl-2 R | CAGATGCCGGTTCAGGTACTCAGTC |
| Mm Cdkn1a F | GCAGATCCACAGCGATATC |
| Mm Cdkn1a R | CAACTGCTCACTGTCCACGG |
| Cre F2 | CGGTCTGGCAGTAAAAACTAT |
| Cre R2 | CAGGGTGTTATAAGCAATCCC |
| Mm Ppia F | CCTTGGGCCGCGTCTCCTT |
| Mm Ppia R | CACCCTGGCACATGAATCCTG |
| Mm Fbxo7 F | TCACACGGCAAGTACTGAAC |
| Mm Fbxo7 R | GGCCAGGACAGAATGAAC |
| Mm Gadd45a F | GAGGGACTCGCACTTGCAATATGAC |
| Mm Gadd45a R | CAGGATGTTGATGTCGTTCTCGCAG |
| Mm Gapdh F | TGTGTCCGTCGTGGATCTGA |
| Mm Gapdh R | CCTGCTTCACCACCTTCTTG |
| Mm Sfn F | GTGTGTGCGACACCGTACT |
| Mm Sfn R | CTCGGCTAGGTAGCGGTAG |
